# Explainable IAOA-CNN-CBAM-SVR model for predicting air consumption of auxiliary nozzles with limited sample size

**DOI:** 10.1371/journal.pone.0351109

**Published:** 2026-06-05

**Authors:** Min Shen, Yongbo Cao, Xiaoshuang Xiong, Zhen Wang, Lianqing Yu, Xuezheng Yang, Yongfa Lv

**Affiliations:** 1 Three-dimensional Textile Hubei Engineering Research Center, Wuhan Textile University, Wuhan, China; 2 School of Mechanical Engineering and Automation, Wuhan Textile University, Wuhan, Hubei, China; 3 Rifa Textile Machinery Co., Ltd, Liaocheng, Shandong, China; Newcastle University, UNITED KINGDOM OF GREAT BRITAIN AND NORTHERN IRELAND

## Abstract

Air-jet looms are energy-intensive machines, with auxiliary nozzles accounting for nearly 80% of the total compressed air consumption. However, accurate prediction and visual analysis of nonlinear air consumption remain challenging due to limited training data and the poor interpretability of deep learning models. To address these issues, this study proposes a hybrid CNN-CBAM-SVR model optimized by an Improved Archimedes Optimization Algorithm (IAOA). Comparative experiments show that the IAOA-CNN-CBAM-SVR model achieves the lowest root mean square error (RMSE) of 0.6575, and the highest coefficient of determination (R^2^) of 0.9941, outperforming SVR, CNN, and CNN-SVR models. Furthermore, the contributions of nozzle structural parameters to air consumption are visually illustrated using the Shapley Additive ExPlanations (SHAP) method. The findings provide a robust and interpretable model for optimizing auxiliary nozzles design and improving energy efficiency in air-jet looms.

## 1. Introduction

Air-jet looms are the most productive shutterless weaving machine due to its automation and production efficiency. The weft insertion utilizes a main nozzle along with multiple auxiliary nozzles, which are precisely controlled blowing times and pressure to ensure the weft across the shed [1]. In general, an air jet loom requires 40-100m^3^/h of air at 0.6MPa pressure, depending on the fabric type and yarn insertion velocity [2]. The auxiliary nozzles take up 80% of the air volume supplied by an air compressor, rustling in significant air and electricity usage during the operation of air jet looms in the factory [3]. Reducing outlet diameter of auxiliary nozzle decreased air consumption by 21%. As a result, a textile factory operating 202 looms could save 240,000 €per year in electricity expenditure without reequipment additional investment. This highlights an urgent need for textile manufacturers to reduce energy consumption in air jet looms.

Despite the growing demand for energy-efficient weaving technologies, accurately predicting the air consumption of auxiliary nozzles remains a challenging task. The relationship between nozzle structural parameters and air consumption is highly nonlinear and influenced by multiple interacting variables. Traditional experimental or simulation approaches are often time-consuming and expensive when exploring a large design space. With the rapid development of artificial intelligence, machine learning and deep learning techniques have demonstrated strong potential in solving complex nonlinear regression problems. However, traditional machine learning models often struggle with high-dimensional nonlinear relationships, while deep learning models typically require large training datasets, which are often unavailable in industrial applications. Furthermore, the hyperparameter tuning process of deep learning models is computationally expensive and may lead to suboptimal solutions when using conventional optimization strategies. In addition, many deep learning models operate as “black boxes”, making it difficult to interpret how structural parameters influence the predicted air consumption. These challenges motivate the development of an accurate, efficient, and interpretable prediction framework suitable for limited sample size conditions.

To address these challenges, this study proposes an explainable hybrid CNN-CBAM-SVR framework optimized by an Improved Archimedes Optimization Algorithm (IAOA) to predict the air consumption of auxiliary nozzles under limited training samples. The main contributions of this research are summarized as follows:

(1) A hybrid CNN-CBAM-SVR model is developed, in which the convolutional neural network (CNN) integrated with the Convolutional Block Attention Module (CBAM) enhances the extraction of critical structural features from the auxiliary nozzle, thereby improving prediction accuracy. By incorporating the CBAM attention mechanism, the proposed framework can emphasize important features and suppress irrelevant information, leading to more effective feature representation.
(2) The framework integrates the Support Vector Regression (SVR) with the CNN-CBAM module to improve generalization and accelerate convergence, particularly training on small datasets. Previous prediction models frequently suffer from overfitting or unstable performance when the available training data are limited. By combining the feature extraction capability of CNN-CBAM with the strong generalization ability of SVR, the proposed hybrid model achieves more robust prediction performance under limited sample size conditions.
(3) An Improved Archimedes Optimization Algorithm (IAOA) is proposed to autonomously optimize the hyperparameters of the hybrid model. The IAOA incorporates good point set initialization, adaptive feedback factors, and a Lévy rotation transformation strategy to enhance search efficiency and prevent premature convergence. Traditional hyperparameter optimization methods, such as grid search or basic heuristic algorithms, often suffer from inefficient exploration and a tendency to converge to local optima. The proposed IAOA improves global search capability and convergence stability, thereby enabling more effective hyperparameter tuning for the hybrid model.
(4) Model interpretability is achieved using Shapley Additive Explanations (SHAP), which quantitatively reveals the contribution of each nozzle structural parameter to the predicted air consumption, providing insights for design optimization. Most existing studies treat machine learning models as black boxes and provide limited interpretation of how structural parameters influence prediction outcomes. By introducing SHAP analysis, the proposed framework offers a transparent interpretation of model predictions and facilitates a deeper understanding of the relationships between nozzle structural parameters and air consumption.

### 1.1. Air consumption studies in air-jet looms

Extensive effort has been devoted to investigating auxiliary nozzle in air jet looms for save electricity and reduce carbon emissions. Pioneer, Adanur et al. [4] claimed that the air consuption was reduced by maintaining the air pressure of the auxiliary nozzle and lowering solenoid valve drive time. Grassi et al. [5] designed a kind of convergent auxiliary nozzle that reduces energy consumption by up to 30% in air-jet weaving systems. However, previous researchers investigated air consumption of the air jet loom based on experiment and qualitative analysis. Currently, there is a growing research interest in investigation the relationship between the air consumption and structural features of auxiliary nozzles. Haq and Hossian [6] investigated critical factors influence the air consumption such as, structure of nozzle, duration of blowing, and arriving time. Hossain et al. [7] found that air supply pressure could be reduced by adjusting the position of auxiliary nozzle. Zegan et al. [8] investigated the relationship between air pressure of auxiliary nozzle and loom speed through response surface methods for save energy. Adams et al. [9] analyzed the air pressure, loom speed on the air usage of the air jet loom, indicating that the auxiliary nozzles are intricately linked to air consumption. Ning et al. [10] had proposed a convolutional network combined a self-attention mechanism model for forecasting the air consumption of auxiliary nozzle with an elliptical exit. However, the ML model ofter lack sufficient capability for high-dimensional nonlinear regression anlysis, while deep learnning requires a substantial sample size to assure prediction accuracy. Moreover, the impact of structural parameters of the auxiliary nozzle on air cosnumption has not yet been clearly visuallized or interpreted.

### 1.2. Machine learning and hybrid prediction models

Owing to the rapid development of artificial intelligence, the utilization of machine learning techniques, including support vector regression (SVR) [11], artificial neural network (ANN) [12], as well as deep learning approaches, particularly convolutional neural network (CNN) [13], and Transformer [14], have arisen to address nonlinear regression. Despite the potential of artificial technique application, it is essential to acknowledge that the ML model hinges on deficiency for high-dimensional nonlinear systems, and DL requires a substantial sample size to assure prediction accuracy. To mitigate the limitations of individual ML or DL models, researchers have developed hybrid architectures that integrate deep learning feature extraction with regression models. Safavi et al. [15] have developed a CNN-XGBoost model to predict battery life in energy storage systems. Ghimire et al. [16] have developed a novel hybrid convolutional neural network (CNN) and support vector regression (SVR) model for estimating solar radiation in Queensland. Rastegar et al. [17] provided a hybrid CNN-SVR model that can derive characteristic features from electrocardiogram (ECG) and photoplethysmography (PPG) signals to accurately predict systolic blood pressure (SBP) and diastolic blood pressure. Özbay et al. [18] constructed an integrated CNN-SVR framework for prediction hydropower generation. Botrugno et al. [19] proposed a CNN-SVR model for accurately predicting arterial blood pressure based on multiwavelength Photoplethysmography (PPG) signals. Compared to standalone machine learning or deep learning models, hybrid model can improve the accuracy and generalization performance of regression prediction. However, such hybrid models typically require extensive hyperparameter tuning to achieve optimal accuracy.

### 1.3. Optimization algorithms for hyperparameter tuning

In response to the need for optimization hyperparameter in ML and DL model, numerous scholars have devised various heuristic optimization algorithms [20]. The category encompasses natural evolutionary algorithms, including Genetic Algorithm (GA) [21], the Moss Growth Optimization (MGO) [22], among others. There have also been heuristic optimization algorithms inspired by swarm intelligence, such as Gray Wolf Optimization algorithm (GWO) [23], Whale Optimization Algorithm (WOA) [24], Sparrow search algorithm (SSA) [25] and so on. Sun et al. [23] employed GWO to obtain the hyperparameters of the Bidirectional Long Short-term memory network (Bi-LSTM) for short-term wind power prediction. Cui et al. [24] proposed a hybrid CNN-CBAM-LSTM model incorporating a Whale Optimization Algorithm (WOA) to automatically determine the hyperparameters of the hybrid model. Tu et al. [25] developed a hybrid CNN-SVR model using an improved Sparrow search algorithm (ISSA) optimization hyperparameter of the model. However, the dependence of swarm intelligence algorithms on group collaboration restricts their global search efficacy in high-dimensional space, and they cannot ensure correctness and robustness in solving complicated issues due to insufficient mathematical theoretical support.

Furthermore, physics-based metaheuristic optimization algorithms incorporate physics rules into optimization processes, often demonstrating superior outcomes over natural evolutionary or swarm-intelligence algorithms in engineering applications [26]. Notable recent development in this domain includes the Fick’s Law Algorithm (FLA) [27], Tornado Optimizer with Coriolis Force (TOC) [28], and Archimedes optimization algorithm (AOA) [29]. The AOA is inspired by the buoyancy-driven motion of objects immersed in a fluid, which features a simple conceptual framework and ease of implementation. However, it is prone to premature and may suffer from slow convergence rates, particularly in later optimization stages, leading to long computational times when seeking high quality solutions.

The remainder of this paper is organized as follows: Section 2 briefly introduces the experimental methodology for acquiring the air consumption dataset. Section 3 introduces the establishment of a hybrid model integrating SVR machine learning and CNN-Attention deep learning to enhance the efficiency of high-dimensional nonlinear regression with limited training samples. In section 4, an improved Archimedes Optimization Algorithm is proposed for automatically optimizing the hyperparameter of the hybrid model. Section 5 presents the SHAP-based interpretability analysis, followed by the conclusions in Section 6.

In summary, this study aims to construct an explainable hybrid IAOA-CNN-CBAM-SVR model optimized by the improved Archimedes Optimization Algorithm to predict the air consumption of auxiliary nozzles. The results verify that the proposed model not only achieves accurate prediction but also provides interpretable insights into the influence of nozzle structure on energy consumption.

## 2. Experimental setups

**Fig 1:**
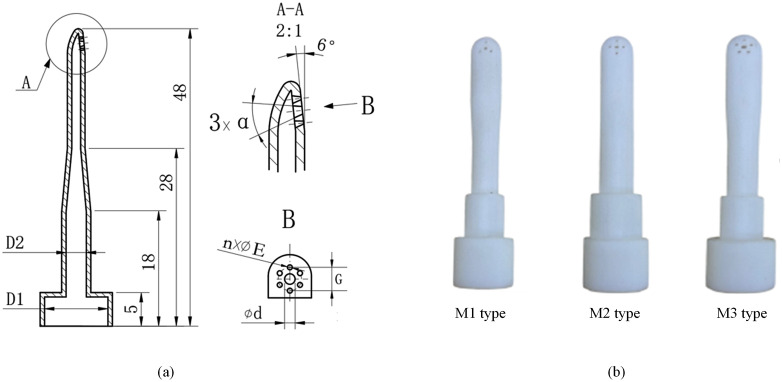
Auxiliary nozzle in the air jet loom (a) structural parameters (b) 3D printing auxiliary nozzle.

In the air jet loom, the auxiliary nozzle is used to accelerate air flow speed in the profiled reed. When air leaving the nozzle mixes with the still air, it generates a subsonic jet to propel the weft through a reed. Structural parameters of auxiliary nozzle have a significant impact on air consumption. The structural parameters include the outlet diameter of the circular hole (d *mm*), inlet diameter (D1 *mm*), straight pipe diameter (D2 *mm*), and conical degree (*α rad*), as illustrated in Fig 1. The rapidly developing 3Dprinting technology has been applied in the manufacturing of complex materials. It has a high degree of flexibility compared to traditional manufacturing processes and can achieve rapid manufacturing of complex structure without need for molds. Different types of auxiliary nozzle were manufactured using 3D printing technology. Three types of auxiliary nozzle 3D printing models named M1, M2 and M3 are showing in Fig 1.

**Fig 2:**
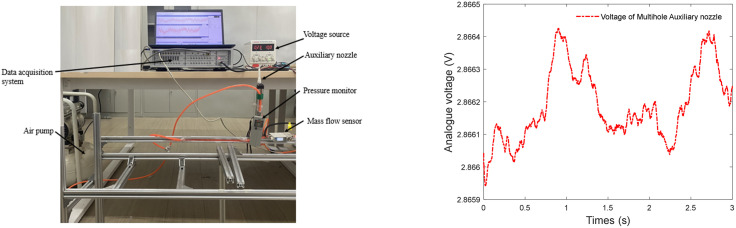
Air flow rate acquisition system.

The key metric of air consumption is the flow rate through an auxiliary nozzle. An air flow meter is a device which measures the amount of air volume through the auxiliary nozzle. The test configuration comprises a Siemens LMS data acquisition system (SCADAS), a MEMS flow sensor (model AFM0725H00S), an auxiliary nozzle, a pressure monitor and an air pump. The data acquisition software is SimcenterTestlab2021.2. All experimental devices are shown in Fig 2.

## 3. Hybrid CNN-CBAM-SVR model

### 3.1. Convolution neural network

Convolutional neural network (CNN) is a category of feedforward neural network in deep learning that has excellent extract feature capabilities [30]. A two-dimensional convolutional neural network(2D-CNN) extracts structural features of the auxiliary nozzle. The CNN architecture consists of convolutional layers alternating with pooled layers, followed by dense layers. The CNN is core of the extract input feature, which employs the concept of weight sharing and the use of numerical filters to derive characteristics from the raw input data.

### 3.2. Convolutional block CBAM module

**Fig 3:**
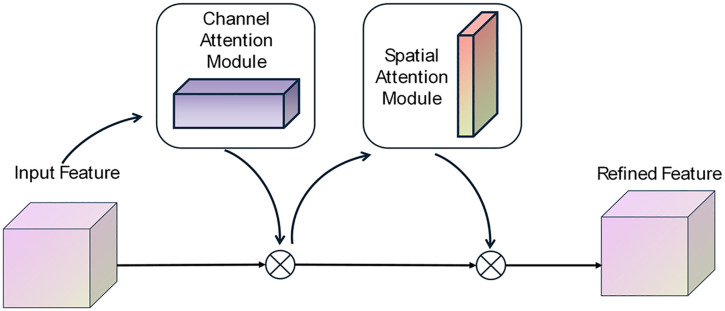
The structure of CBAM.


The Convolutional Block Attention Module (CBAM) is a novel attention mechanism capturing fine grained input feature in dual dimensions [31]. This study utilizes CNN as baseline module integrating CBAM to improve prediction accuracy with small size samples. The CBAM has two sequential channel and spatial sub-modules, as shown in Fig 3. It can utilize max and average pooling operation to capture both maximum and average response within the input feature. The formula for CBAM is as follows:

$$\begin{array}{c} M_{ch}=\sigma \left(f_{mlp}(A\left(F_{I}\right))+f_{mlp}\left(M\left(F_{I}\right)\right)\right) \end{array}$$ (1)

$$\begin{array}{c} M_{sp}=\sigma \left(f_{cov}\left(C_{oncat}(A\left(F_{I}\right),M\left(F_{I}\right))\right)\right) \end{array}$$ (2)

$$\begin{array}{c} M_{fi}=M_{sp}\left(M_{ch}⊗F_{I}\right)⊗\left(M_{ch}⊗F_{I}\right) \end{array}$$ (3)

Where, σ is sigmoid function, *f*_*mlp*_ is multilayer perception, *f*_*cov*_ is the convolutions, *A* and *M* are average pooling and maximum pooling, respectively. ⊗ indicates the element wise multiplication.

### 3.3. Support vector regression

Support vector regression (SVR) is a regression approach derived from statistical learning theory. The SVR transforms a nonlinear problem into a linear problem by introducing a kernel function to map the original data into a high-dimensional feature space [13]. In high-dimensional space, SVR approaches the objective function by minimizing the structural risk, which can effectively model the complex relationship.

The support vector regression formula is as follows:


$$\begin{array}{c} f\left(x\right)=w_{i}\phi_{i}\left(x\right)+b \end{array}$$ (4)

In the above formula, *i*=1, 2,..., n, n is the number of samples; *x* is the input vector; *w*_*i*_ is the weight vector; *b* is offset; *ϕ*_*i*_(*x*) is an eigenvector that maps the input vector *x* to a high-dimensional eigenspace. The optimization equation of support vector regression model is as follows:


$$\begin{array}{c} \begin{array}{c} minimize\{\frac{\text{1}}{\text{2}}∥w{∥}^{\text{2}}+C\sum_{i=1}^{n}(\xi_{i}+\overset{*}{\underset{i}{\xi }})\}\\ s.t:\left\{ \begin{array}{c} @l@y_{i}-f\left(x_{i}\right)-b\leq \varepsilon +\xi_{i}\\ f\left(x_{i}\right)-y_{i}\leq \varepsilon +{\xi_{i}}^{*}\;,(i=1,2,\cdots ,n)\;\\ \xi_{i}\geq 0,\overset{*}{\underset{i}{\xi }}\geq 0 \end{array}\right. \end{array} \end{array}$$ (5)

Where *ξ*_*i*_ and ξ* i are relaxation variables; *C* is a regularization parameter, which is used to prevent the model from overfitting. The smaller the value of *C*, the lower the penalty on the model and the stronger the generalization ability. *ε* is a loss function parameter.

The final model equation is as follows:


$$\begin{array}{c} y=f\left(x\right)=\sum_{i=1}^{n}(\overset{*}{\underset{\text{1}}{\overset{―}{a}}}-{\overset{―}{a}}_{\text{1}})k\left(x_{i},x\right)+b \end{array}$$ (6)

Where $\overset{*}{\underset{\text{1}}{\overset{―}{a}}}$ and ${\overset{―}{a}}_{\text{1}}$ are Lagrange coefficients; *k* is the kernel function. Here, Gaussian kernel is selected as its kernel function, as shown in Eq. (7).


$$\begin{array}{c} k\left(x_{i},x\right)=exp{\left(-\frac{\|x-x_{i}\|}{2\sigma^{\text{2}}}\right)}^{\text{2}} \end{array}$$ (7)

In Eq. (7), the variable *σ* indicates the bandwidth of the Gaussian radial basis kernel function.

**Fig 4:**
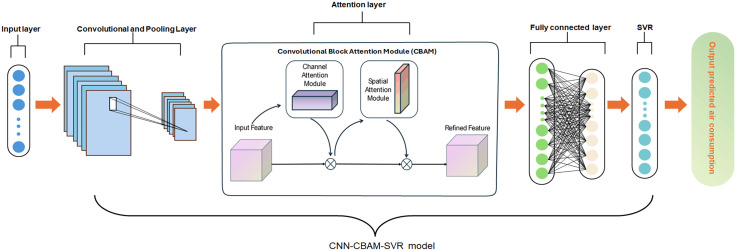
The structure of hybrid CNN-CBAM-SVR model.

In this section, a hybrid CNN-CBAM-SVR model is proposed, as illustrated in Fig 4. The CNN first extracts high dimensional input features of the auxiliary nozzle. Further, the CBAM mechanism adaptively recalibrates weights to enhance input features. Finally, the processed features are fed through a fully connected layer into a SVR model to perform regression analysis air consumption of the auxiliary nozzle.

## 4. Optimization algorithm

### 4.1. Archimedes optimization algorithm

The AOA assigns four attributes-volume, density, acceleration and position to individual, and continuously updating these attributes during the iterative process to attain global optimization of the fitness function. The AOA algorithm is divided into the exploration stage and the development stage based on whether collisions occur or not. If there is a collision, it is the exploration stage. While the absence of collision indicates the transition to the development stage.

To achieve a smooth transition between the two stages, the transfer operator (*TF*) is introduced, and its calculation formula is as follows:


$$\begin{array}{c} TF=exp\left(\frac{t-t_{max}}{t_{max}}\right) \end{array}$$ (8)

Where, *t* and *t*_*max*_ denote the current iteration number and the maximum iteration number respectively.

During the initialization phase, the positions of all objects are initialized using the following formula:


$$\begin{array}{c} \left\{ \begin{array}{c} @l@d_{i}=rand\\ v_{i}=rand\\ a_{i}=l+rand\times (u-l)\\ x_{i}=l+rand\times (u-l) \end{array}\right. \end{array}$$ (9)

Among them, *d*_*i*_, *v*_*i*_, *a*_*i*_, and *x*_*i*_ represent the density, volume, acceleration, and position of the *i*-th individual respectively. *i*=1, 2, 3,... *N* and rand are random numbers on [0,1], and *u* and *l* represent the upper and lower limits of the solution space respectively.

Immediately afterwards, update the density and volume:


$$\begin{array}{c} \left\{ \begin{array}{c} @l@\overset{t+1}{\underset{i}{d}}=\overset{t}{\underset{i}{d}}+rand\times (d_{best}-\overset{t}{\underset{i}{d}})\\ \overset{t+1}{\underset{i}{v}}=\overset{t}{\underset{i}{v}}+rand\times (v_{best}-\overset{t}{\underset{i}{v}}) \end{array}\right. \end{array}$$ (10)

In the formula, *d*_*best*_ and *v*_*best*_ respectively represent the density and volume of the optimal individuals in the population.

Finally, update the acceleration and position. When *TF*≤0.5, the exploration stage is carried out:


$$\begin{array}{c} \left\{ \begin{array}{c} @l@\overset{t+1}{\underset{i}{a}}=\frac{d_{mr}+v_{mr}\times a_{mr}}{\overset{t+1}{\underset{i}{d}}\times \overset{t+1}{\underset{i}{v}}}\\ \overset{t+1}{\underset{i}{x}}={x_{i}}^{t}+C_{\text{1}}rand\cdot \overset{t+1}{\underset{i-norm}{a}}\cdot D^{t+1}\times (x_{rand}-{x_{i}}^{t}) \end{array}\right. \end{array}$$ (11)

When *TF* >0.5, the exploitation stage is carried out:


$$\begin{array}{c} \left\{ \begin{array}{c} @l@\overset{t+1}{\underset{i}{a}}=\frac{d_{best}+v_{best}\times a_{best}}{{d_{i}}^{t+1}\times \overset{t+1}{\underset{i}{v}}}\\ \overset{t+1}{\underset{i}{x}}=\overset{t}{\underset{best}{x}}+FC_{\text{2}}rand\cdot \overset{t+1}{\underset{i-norm}{a}}\cdot D^{t+1}\times (T\cdot x_{best}-{x_{i}}^{t}) \end{array}\right. \end{array}$$ (12)

In Eqs. (11) to (13), *C*_1_ and *C*_2_ are constants; *T* increases with time and is defined as *T* = *C*_3_ × *TF*, where *C*_3_ is a constant. *d*_*mr*_, *v*_*mr*_ and *a*_*mr*_ respectively represent the density, volume and acceleration of a certain random individual, *x*_*rand*_ represents the position of the randomly selected individual, *a*_*best*_ and *x*_*best*_ respectively represent the acceleration and position of the optimal individual in the population. *a*_*i-norm*_ represents the normalized acceleration; *D*^*t+1*^ is the density factor; *F* is a random direction variable. Each operator is:


$$\begin{array}{c} \overset{t+1}{\underset{i-norm}{a}}=u_{a}\times \frac{\overset{t+1}{\underset{i}{a}}-min\left(a_{i}\right)}{max\left(a_{i}\right)-min\left(a_{i}\right)}+l_{a} \end{array}$$ (13)

$$\begin{array}{c} D^{t+1}=exp\left(1-\frac{t}{t_{max}}\right)-\frac{t}{t_{max}} \end{array}$$ (14)

$$\begin{array}{c} F=\left\{ \begin{array}{c} @l@\;1\;,if\;p\leq 0.5\\ -1\;,if\;p>0.5 \end{array}\right. \end{array}$$ (15)

Where, *P*=2×*rand*-*C*_4_, where *C*_4_ is a constant. *u*_*a*_ and *l*_*a*_ are the upper and lower limits of the acceleration normalization range respectively, and the common values are *u*_*a*_ =0.9 and *l*_*a*_ =0.1.

### 4.2. Improved AOA algorithm

Although the AOA has a simple structure, few parameters, strong search ability, and good convergence performance. However, it suffers low population diversity, weak correlation between optimization range and process, and high risk of falling into local optima. To address these problems, an improved AOA(IAOA) is proposed. Three strategies are introduced in IAOA. Firstly, a good nodes set initialization is used to generate a high-quality initial population. Secondly, adaptive feedback adjustment weight is used to enhance exploitation and exploration. Finally, the Lévy rotation transformation strategy is adopted to enhance global search capability to avoid getting stuck in local optima. The hyperparameter of the CNN-CBAM-SVR combination model was optimized using IAOA.

#### 4.2.1. Good nodes set population initialization.

Considering that the diversity of initial population greatly affects the convergence speed and accuracy of AOA algorithms. The AOA can’t guarantee population diversity based on random initialization. Thereby, incorporating good point set initialization to improve the uniformity of the population’s distribution in the solution space, enhancing the global exploration ability. The optimal point set method makes the individuals more evenly distributed in the search space than random initialization.

#### 4.2.2. Adaptive feedback adjustment factor.

To improve the problems that AOA is prone to fall into local optimum and the decline of population diversity in the later iterations, this paper introduces an adaptive feedback moderating factor. This method utilizes the adaptive feedback adjustment factor to dynamically adjust the density factor, enabling individuals to flexibly change according to their own status and the population environment. Thereby the local development ability is enhanced, maintaining population diversity. For instance, the success value *Q* (*i*, *t*) of individual *i* at iteration *t* for a minimization problem is defined as follows:


$$\begin{array}{c} Q\left(i,t\right)=\left\{ \begin{array}{c} @l@1\;fit\left(pbest^{i}\left(t\right)\right)<fit\left(pbest^{i}\left(t-1\right)\right)\\ 0\;fit\left(pbest^{i}\left(t\right)\right)=fit\left(pbest^{i}\left(t-1\right)\right)\; \end{array}\right. \end{array}$$ (16)

Where *pbest*^*i*^(*t*) represents the historical best position of individual *i* at iteration t and *fit* (·) is the fitness function. The population success rate (*P*_*s*_) is determined by the success value *Q* (*i*, *t*) of individual *i*, defined as the ratio of the number of evolutionarily successful individuals corresponding to the individual’s historical best position to the population size.


$$\begin{array}{c} P_{s}=\frac{\text{1}}{N}\sum_{i=1}^{N}Q\left(i,t\right) \end{array}$$ (17)

Where *N* represents the population size.

In the early stage of the algorithm, individuals are widely distributed in the solution space and have a relatively high evolutionary success rate, indicating that the current optimal individual has a strong guiding role. As the iteration progresses, the population gradually converges and the success rate of evolution decreases, indicating that more individual information needs to be retained to enhance the local search ability. In this paper, an adaptive feedback adjustment factor was adopted to dynamically balance the global exploration and local development capabilities. Its definition is as follows:


$$\begin{array}{c} D^{t+1}=2\cdot exp(1-\frac{t}{t_{max}})-\frac{t}{t_{max}}\times (\alpha \cdot P_{s}(t)+\beta \cdot F_{i}) \end{array}$$ (18)

Where the parameter *α* denotes the coefficient of importance of evolutionary success of the population, *β* denotes the importance coefficient of this fitness factor*,* and *F*_*i*_ defined as:


$$\begin{array}{c} F_{i}=\lambda^{-(f(X_{i})-f_{min}+\varepsilon )/(f_{max}-f_{min}+\varepsilon )} \end{array}$$ (19)

Here, *F*_*i*_ represents the normalized fitness coefficient of the *i*-*th* individual; *λ* is a decay factor; and *ε* is a very small rational number to prevent division by zero. The adaptive feedback adjustment factor dynamically adjusts according to changes in fitness; if an individual’s fitness approaches the optimal fitness, the feedback factor maintains the original density factor characteristics, accelerating the convergence rate. For individuals with lower fitness, the adaptive feedback factor approaches its maximum value, preserving more individual information and ensuring the algorithm’s local exploitation capability.

#### 4.2.3. Lévy rotation transformation strategy.

To maintain population diversity and enhance individual exploration capabilities in later iterations of AOA. A Lévy rotation transformation strategy is proposed. This strategy applies a rotational transformation around the optimal individual as the center, with the Lévy step size as the radius, to increase both population diversity and convergence speed. The Lévy rotation transformation operator is defined as follows:


$$\begin{array}{c} \overset{t}{\underset{i}{x}}=x_{best}+\frac{s}{n∥\overset{t}{\underset{i}{x}}|{|}_{\;2}}R_{r}\overset{t}{\underset{i}{x}} \end{array}$$ (20)

Where, *R*_*r*_∈*R*_n*n_ is a random matrix with elements taking values uniformly distributed between [-1, 1]; ‖**·**‖ is a vector 2-parameter; *s* is a rotation factor combining Lévy; by principle the position can be rotated to any position with radius *s*, which can be used for controlling the search range of the solution, and its expression is as follows:


$$\begin{array}{c} s=0.01\times \frac{\mu }{|\nu {|}^{1/\beta }}(\overset{t}{\underset{i}{x}}-x_{best}) \end{array}$$ (21)

Where *μ* and *ν* follow normal distributions, *μ*~*N* (0, *σ2 u*), *ν*~*N* (0, *σ2 v*).


$$\begin{array}{c} \sigma_{\mu }={\left\{\frac{\Gamma (1+\beta )\times sin\left(\frac{\pi \beta }{\text{2}}\right)}{\Gamma \left[\frac{\left(1+\beta \right)}{\text{2}}\right]\beta \times {\text{2}}^{\frac{\beta -1}{\text{2}}}}\right\}}^{1/\beta } \end{array}$$ (22)

The search radius’s size is determined by the value of *β*; the higher the value of *β*, the more robust the localization ability.

The Lévy rotation transformation operator rotates individuals at a specific angle to maximize the avoidance of duplicate individuals. By leveraging the Lévy step’s characteristic alternation between short and long steps, this strategy broadens the search scope of the population, assisting the algorithm in escaping local optima when necessary to enhance search performance. To balance exploration depth and breadth, the evolution probability of each individual is calculated based on its fitness value, as shown in the equation below. The lower the quality of an individual’s feasible solution, the higher the probability it will undergo the Lévy rotation transformation. Additionally, a greedy selection is used to update solutions, maintaining elite individuals while promoting exploration ability.


$$\begin{array}{c} p_{i}=\frac{fit}{\sum_{i=1}^{N}fit_{i}} \end{array}$$ (23)

Where, *p*_*i*_ is the selection probability; *fit*_*i*_ is expressed as.


$$\begin{array}{c} fit=\left\{ \begin{array}{c} @l@\frac{\text{1}}{1+f_{i}},\;f_{i}\geq 0\\ 1+\left|f_{i}\right|,\;f_{i}<0 \end{array}\right. \end{array}$$ (24)

Where, *f*_*i*_ is the fitness value of the *i*-*th* individual.

### 4.3. Implementation details

**Fig 5:**
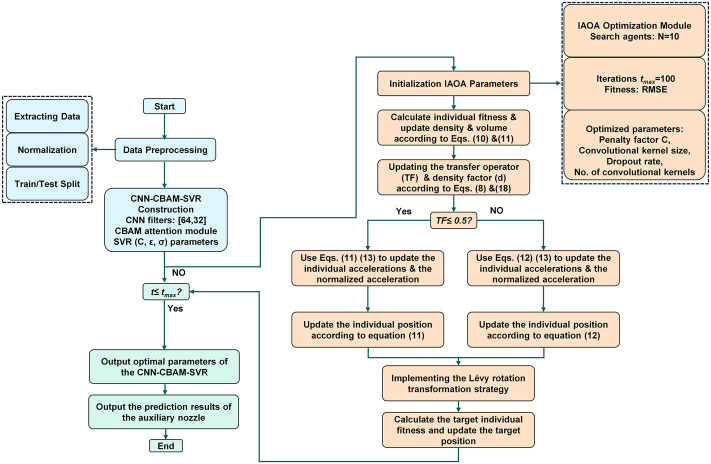
Flowchart of IAOA Optimized the hybrid CNN-CBAM-SVR Model Process.

This section describes the application of the IAOA algorithm to optimize the hyperparameters of hybrid CNN-CBAM-SVR model. The flowchart is illustrated in Fig 5. In the hybrid CNN-CBAM-SVR model, the number of output channels of the convolutional layer, the size of the convolution kernels, dropout rate, and the penalty parameters C, and the relaxation coefficient ε are taken as optimized individuals. The root mean square error (RMSE) was used as the fitness function during the IAOA iteration process.

### 4.4. Model evaluation metrics

To verify the prediction accuracy of the hybrid model, five different evaluation indicators were adopted to assess the effectiveness of the proposed prediction model. These include the coefficient of determination (*R²*), root mean square error (*RMSE*), Theil inequality coefficient (*TIC*), mean absolute error (*MAE*), and Willmott’s index of agreement (*WIA*). Their respective calculation formulas are as follows:


$$\begin{array}{c} R^{\text{2}}=\text{1}-\frac{\sum (y_{i}-{\hat{y}}_{i}{)}^{\text{2}}}{\sum (y_{i}-\bar{y}{)}^{\text{2}}} \end{array}$$ (25)

$$\begin{array}{c} RMSE=\sqrt{\frac{\text{1}}{N}\sum_{i=\text{1}}^{N}(y_{i}-{\hat{y}}_{i}{)}^{\text{2}}} \end{array}$$ (26)

$$\begin{array}{c} TIC=\frac{\sqrt{\frac{\text{1}}{n}\sum_{i=1}^{n}{\left({\hat{y}}_{i}-y_{i}\right)}^{\text{2}}}}{\sqrt{\frac{\text{1}}{n}\sum_{i=1}^{n}{\left({\hat{y}}_{i}\right)}^{\text{2}}}+\sqrt{\frac{\text{1}}{n}\sum_{i=1}^{n}{\left(y_{i}\right)}^{\text{2}}}} \end{array}$$ (27)

$$\begin{array}{c} MAE=\frac{\text{1}}{N}\sum_{i=\text{1}}^{N}\left|y_{i}-{\hat{y}}_{i}\right| \end{array}$$ (28)

$$\begin{array}{c} WIA=1-\frac{\sum_{i=1}^{n}{\left({\hat{y}}_{i}-y_{i}\right)}^{\text{2}}}{\sum_{i=1}^{n}{\left(\left|{\hat{y}}_{i}-{\overset{―}{y}}_{i}\right|+\left|y_{i}-{\overset{―}{y}}_{i}\right|\right)}^{\text{2}}} \end{array}$$ (29)

In the equations above, $y_{i}$ represents the actual value, ${\hat{y}}_{i}$ denotes the predicted value, and *N* signifies the number of samples.

## 5. Results and discussion

### 5.1. Data description

The air flow of each auxiliary nozzle was measured using an LMS data acquisition system equipped with a calibrated flow sensor. The air flow was recorded in units of L/min, and the sensor was calibrated according to the manufacturer’s standard procedure before the experiments to ensure measurement accuracy. Each nozzle configuration was measured five times, and the average value was taken as the result to reduce random measurement errors. The original voltage signal collected by the LMS system was filtered using a rectangular window function to reduce noise measurement. Specifically, a moving average filter based on a rectangular window with a window length of 50 sampling points was applied to smooth the signal. The sampling frequency of the data acquisition system was 1 kHz, meaning that each window corresponded to 50 ms of signal duration. The experiment was conducted in a laboratory with a constant temperature of 25°C and a humidity of 35%. To establish the mapping relationship between the structural parameters of the auxiliary nozzle and the air consumption, the Latin Hypercube Sampling (LHS) method was used to generate 110 groups of auxiliary nozzle parameter combinations within the specified parameter ranges. All samples correspond to physically measured experimental data obtained from the test platform. The dataset was divided into training, validation, and testing sets with a ratio of 7:1:2. Among them, the value range of inlet diameter D1 is [4, 8.8], straight pipe diameter D2 is [2, 4.2], exit’s large hole diameter d1 is [0.6, 1.6], exit’s small hole diameter d2 is [0.4, 1.0], and the value range of α is [14°, 40°]. Table 1 presents the air flow data of 12 representative auxiliary nozzle samples selected from the 110 experimental cases.

**Table 1:** Structural parameters and outlet air consumption of multi-circular hole auxiliary nozzle.

Serial number	Inlet diameter D1 (mm)	Straight pipe diameter D2 (mm)	Diameter of the large hole d1 (mm)	Diameter of the small hole d2 (mm)	Conical degree α(°)	Air flow rate (L/min)
1	4.0	2.0	0.6	0.4	40	2.06
2	7.6	3.6	0.8	0.4	40	3.37
3	7.6	2.2	0.9	0.4	40	4.59
4	7.8	4.2	0.9	0.5	28	10.72
5	5.4	4.6	0.9	0.6	24	15.66
6	7.4	5.2	0.9	0.6	20	18.08
7	4.8	3.4	0.8	0.7	22	20.89
8	7.2	4.2	0.8	0.7	22	22.58
9	7.8	5.0	0.8	0.7	22	24.35
10	7.8	4.2	0.8	1.0	34	34.93
11	7.8	5.0	1.0	1.0	30	37.34
12	8.8	4.2	1.6	1.0	14	57.45

As presented in Table 1, a comparative analysis of various auxiliary nozzles indicates that the airflow rate increases dramatically from 2.06L/min to 57.45L/min. The airflow rate varies from 20.89Lmin to 22.58L/min for the No.7 and No.8 auxiliary nozzle. An in-depth analysis of the airflow rates in each group indicates that marginal impact of the change in inlet diameter on the airflow rate. Especially, the variance in airflow rate between No.9 and No.11 auxiliary nozzle requires systematic investigation. The airflow rate varies from 24.35L/min to 37.34L/min, which reveals that the outlet diameter of the auxiliary nozzle is a critical parameter affecting the airflow rate. It also illustrates the strong non-linear relationship between the airflow rates and auxiliary nozzle’s structural parameter.

### 5.2 Experimental configuration environment

All prediction tasks in this study were conducted on a desktop computer running the Windows 10 operating system. The system was equipped with an AMD EPYC 7702 64-Core Processor, 64 GB of RAM, and an NVIDIA GeForce RTX 4080 SUPER GPU, providing sufficient computational resources for model training and evaluation. The proposed hybrid model was implemented in Python 3.9.13 using the PyCharm development environment. The deep learning components were developed based on the PyTorch and Keras frameworks. To improve the stability and convergence of the learning process, the Min–Max normalization technique was applied to scale all input features into the range of [0, 1]. Feature normalization helps prevent large differences in feature magnitudes, which may otherwise lead to unstable gradient updates and reduced prediction accuracy. The proposed hybrid model completes the prediction task within approximately 600 seconds during the training stage, demonstrating its computational feasibility for practical engineering applications.

### 5.3. Optimization hyperparameter of hybrid model

In this paper, the CNN, CNN-CBAM, CNN-SVR and CNN-CBAM-SVR all take the CNN layer as the core. The specific parameters are shown in Table 2. To ensure the consistency of the training process, all models adopt the same settings: the size of the convolution kernel is 2, the number of convolution layers is 2, the number of convolution kernels is 64 and 32 respectively, the optimizer is Adam, the number of training rounds is 100, the learning rate is 0.01, the mini-batch size is 32, and the dropout rate is 0.2. On this basis, the CNN-CBAM and CNN-CBAM-SVR models introduce a spatial CBAM mechanism (with a kernel size of 7 and a reduction ratio of 16) to enhance the models’ ability to focus on the key features of input parameter. However, the CNN and CNN-SVR models do not contain the CBAM module. Furthermore, CNN-SVR and CNN-CBAM-SVR are connected to the SVR regressor after the fully connected layer. By setting the penalty factor 1.0 and the insensitive parameter ε = 0.1, the RBF function σ parameter was adjusted according to data variance.

**Table 2:** Hyperparameter of various models.

Parameter	CNN	CNN-CBAM	CNN-SVR	CNN-CBAM-SVR
Convolutional kernel size	2	2	2	2
No. of convolutional kernels	64/32	64/32	64/32	64/32
No. of convolutional layers	2	2	2	2
Channel CBAM_Input channel	None	64/32	None	64/32
Spatial CBAM_kernel size	None	7	None	7
Reduction ratio	None	16	None	16
Epochs	100	100	100	100
Learning rate	0.01	0.01	0.01	0.01
Mini batch size	32	32	32	32
Dropout rate	0.2	0.2	0.2	0.2
Optimizer	Adam	Adam	Adam	Adam
Penalty factor *C*	None	None	1.0	1.0
RBF functionσ	None	None	0.1	0.1
Epsilonε	None	None	0.1	0.1

To automatically search for the optimal hyperparameters of the CNN-CBAM-SVR model, this paper introduces the IAOA algorithm for optimization, and its configuration is shown in Table 3. This table compares the key parameter configurations of five optimization algorithms (Goose, HHO, WOA, AOA and IAOA) in the CNN hyperparameter optimization task. To ensure the fairness of the optimization process and the consistency of the comparison, all algorithms uniformly set the number of search agents to 10 and the maximum number of iterations to 100. The optimization objective is the 7-dimensional hyperparameter space, covering key network parameters such as the size of the convolution kernel, the number of convolution kernel channels, the Dropout rate, and the SVR penalty factor C, to ensure the comprehensive regulation of the model structure and regularization performance. Among them, the search range of the convolution kernel size of each layer is set as [1,8], the number of output channels is [2^0^, 2^8^], the Dropout rate is [0.1, 0.5], and the penalty factor C is controlled within the range of [0.1, 10]. The setting of these parameter intervals aims to consider both the expressive power of the model and the risk of overfitting, providing a flexible and reasonable tuning space. Overall, each optimization algorithm remains consistent in the design of the search space, with only different optimization strategies. Among them, IAOA is an improved algorithm. Based on the same basic Settings as AOA, it further improves the search efficiency and global convergence ability. This table provides a unified and controllable optimization experimental basis for the subsequent performance comparison of the models.

**Table 3:** The setting of various optimization algorithms.

Parameter	Goose	HHO	WOA	AOA	IAOA
Search agents	10	10	10	10	10
Max iterations	100	100	100	100	100
Dimension of hyperparameter	7	7	7	7	7
Convolutional kernel size	1 ~ 8	1 ~ 8	1 ~ 8	1 ~ 8	1 ~ 8
No. of convolutional kernels	2^0^ ~ 2^8^	2^0^ ~ 2^8^	2^0^ ~ 2^8^	2^0^ ~ 2^8^	2^0^ ~ 2^8^
Dropout rate	0.1 ~ 0.5	0.1 ~ 0.5	0.1 ~ 0.5	0.1 ~ 0.5	0.1 ~ 0.5
Penalty factor C	0.1 ~ 10	0.1 ~ 10	0.1 ~ 10	0.1 ~ 10	0.1 ~ 10

**Fig 6:**
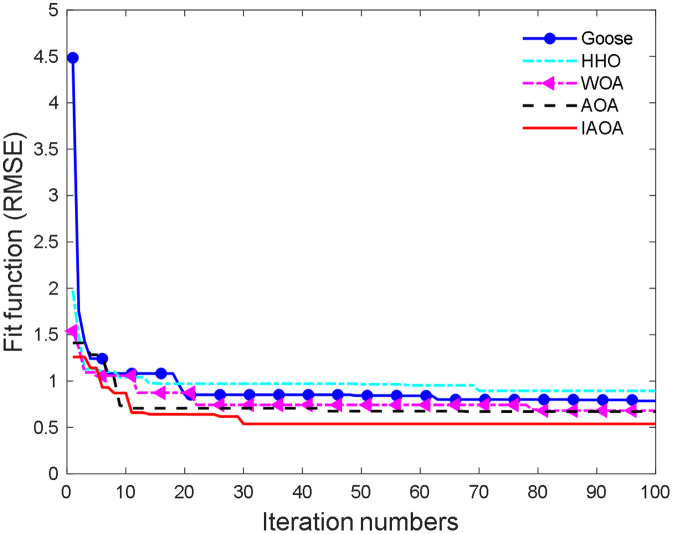
Iteration graph using various optimization algorithms.

To evaluate the performance of various optimization algorithms in the hyperparameter search of the CNN-CBAM-SVR model, this paper compares the IAOA algorithm with the Goose Swarm optimization algorithm (Goose), the Harris Eagle optimization algorithm (HHO), the Whale optimization algorithm (WOA), and the standard AOA algorithm. Fig 6 shows the iteration curve of the fitness function (RMSE) for each optimization algorithm during the iterative process. It can be seen from Fig 6 that the IAOA algorithm begins to converge at the 27th iteration and reaches the minimum fitness value at the 31th iteration, demonstrating excellent convergence speed and search accuracy. In contrast, the AOA algorithm did not achieve convergence until the 40th iteration, while the WOA algorithm began to converge after the 50th generation. Overall, their convergence speeds were relatively slow. Although the HHO and Goose algorithms could also reduce the RMSE in the end, their convergence speeds were very slow and there were still minor fluctuations in the later stages, and the fitness function values were still relatively high, indicating their limited predictive performance. From the comprehensive experimental results, the IAOA optimization algorithm performed the best in terms of iteration speed and accuracy. It not only converged to the optimal solution quickly, but also significantly outperformed other algorithms in terms of fitness function values, which verified its effectiveness and robustness in the hyperparameter optimization task of the CNN-CBAM-SVR model.

### 5.4. Comparative experiment analysis

**Fig 7:**
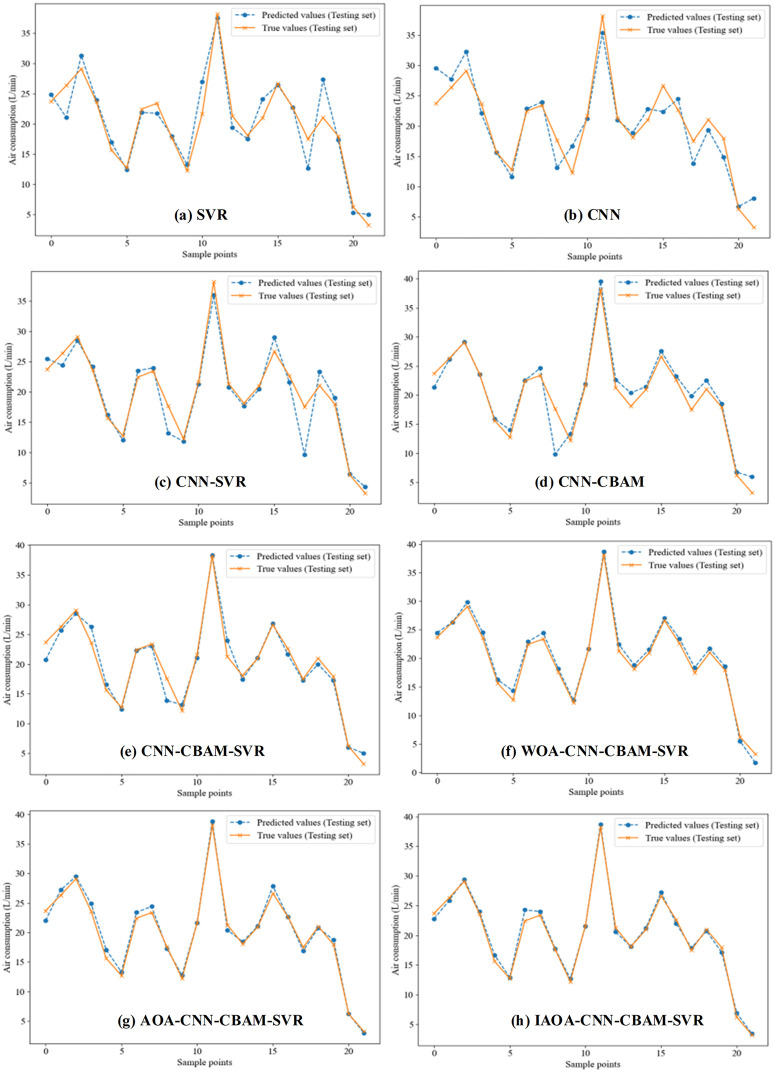
The line chart of prediction air consumption for various model.

Fig 7 plots the line chart of the predicted values and actual values for SVR, CNN, CNN-SVR, CNN-CBAM-SVR models. In Fig 7, the deviation between the actual values and the predicted values can be clearly distinguished for various models. It can be clearly seen from Fig 7 (a)-(b) that most of the predicted values of the SVR and CNN models deviate from the true values, with large fluctuations and prediction errors. As seen in Fig 7 (c)-(d), the CNN-SVR, CNN-CBAM models have some peak predicted values deviated far from the true values, with relatively obvious fluctuation amplitudes. Fig 7(e) illustrates the obvious discrepancies between the prediction valley value and actual values of the CNN-CBAM-SVR model. Fig 7(f)-(g), illustrates that the prediction results of the WOA-CNN-CBAM-SVR model and AOA-CNN-CBAM-SVR models are closely aligned with the true values in the test set, indicating strong fitting capabilities of the hybrid models. The CBAM module effectively enhances the expression ability of key features by incorporating channel and spatial attention mechanisms, hence improving focus on locally important information. As seen in Fig7(h), the last CNN-CBAM-SVR model incorporated with IAOA optimization algorithms exhibited little deviation of the predicted values from the true values. The fluctuation amplitudes between predicted and actual value were minimal. The IAOA implements the Lévy rotation transformation strategy, which improves global search ability. Simultaneously, an adaptive feedback regulatory factor was adopted in IAOA, enhancing convergence efficiency. Consequently, the IAOA-CNN-CBAM-SVR improves both the overall prediction accuracy and calculation efficiency.

**Fig 8:**
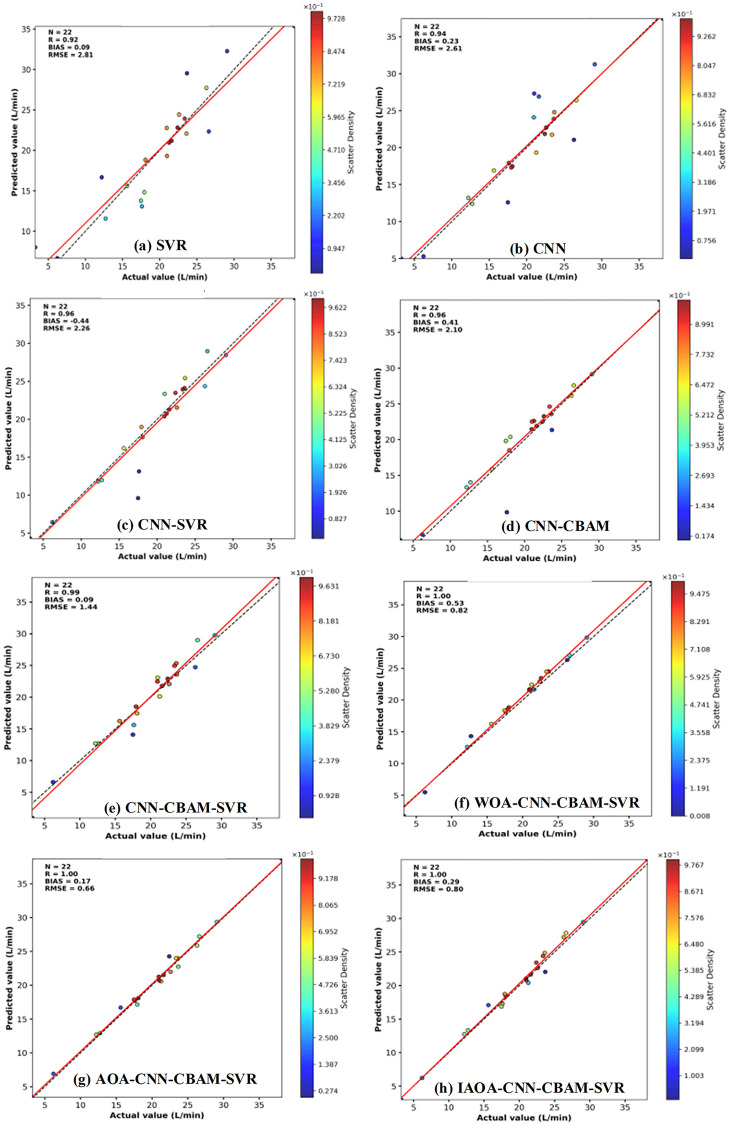
The density scatter plot of various models.

Fig 8(a)-(h) respectively show the density scatter plots of the eight models (SVR, CNN, CNN-SVR, CNN-CBAM, CNN-CBAM-SVR, WOA-CNN-CBAM-SVR, AOA-CNN-CBAM-SVR, and IAOA-CNN-CBAM-SVR) on the test set, which are used to evaluate the degree of fit between the model’s predicted values and the actual values. From Fig.8, it can be clearly seen that the IAOA-CNN-CBAM-SVR model shows a high degree of linear correlation between the predicted values and the actual values, with the scatter density highly concentrated near the diagonal, especially in the middle numerical range, where the fitting effect is the most significant. The deviation index of this model is BIAS = 0.17, indicating that the overall predicted values are slightly higher than the actual values, but the deviation degree is small; RMSE = 0.66, further verifying its strong advantage in error control and demonstrating extremely low prediction errors. In contrast, the scatter distributions of the SVR and CNN models are significantly deviated from the diagonal, with dispersed density, and RMSE exceeds 2.6, indicating poor prediction performance. The combination models of SVR or CBAM mechanisms - CNN-SVR, CNN-CBAM, and CNN-CBAM-SVR - improve the prediction accuracy to some extent, with the scatter density closer to the diagonal, and RMSE is lower than 2.2, demonstrating better prediction ability than the single models. Further, after applying the WOA and AOA optimization algorithms to the CNN-CBAM-SVR model, the scatter plots of WOA-CNN-CBAM-SVR and AOA-CNN-CBAM-SVR models further concentrate towards the diagonal, with RMSE lower than 1.4, verifying the positive role of the optimization algorithms in improving the model’s accuracy. Comprehensive comparison of the scatter plot shapes, and error indicators of each model shows that the IAOA-CNN-CBAM-SVR model performs optimally in terms of fitting accuracy, error control, and generalization ability, demonstrating its outstanding prediction performance and model robustness. Unlike the previous predicted line graph which mainly reflects the point-to-point bias at each sample, the density scatter plot visually presents the correlation and deviation between the predicted values and the true values from the perspective of overall distribution. The line graph emphasizes the model’s tracking ability for trend changes, while the density scatter plot highlights consistency and prediction accuracy. This further verifies that the IAOA-CNN-CBAM-SVR model has comprehensive advantages of high accuracy, and low deviation under the small size sample regression task. The reason is that the CBAM module integrates the channel attention and spatial attention mechanisms, enabling the model to adaptively focus on the most critical feature channels and spatial positions for the output, thereby enhancing the feature expression ability and discriminability of the model.

To verify the accuracy of the proposed model, five evaluation indicators of the test set were compared in Table 4. According to the evaluation index results presented in Table 4, the Theil inequality coefficient (TIC) of the SVR and CNN models exceeds 0.05, and the mean absolute error (MAE) is greater than 1.5. For SVR and CNN model, the prediction error is relatively large. The TIC of the CNN-SVR, CNN-CBAM and CNN-CBAM-SVR models are 0.0533, 0.0486, and 0.0339, respectively. The MAE of IAOA-CNN-CBAM-SVR is 0.5253, which is lower than that of the SVR, CNN and CNN-CBAM models. The integration of the IAOA yields an R^2^ index of 0.9919 for the IAOA-CNN-CBAM-SVR model. The WIA of IAOA-CNN-CBAM-SVR reaches 0.9979, very close to 1, indicating optimal fitting, and the prediction accuracy of the IAOA-CNN-CBAM-SVR model is the highest.

**Table 4:** Comparison of the evaluation indicators of the proposed model.

Model type	R^2^	RMSE	MAE	WIA	TIC
SVR	0.8519	2.8131	2.2301	0.9609	0.0658
CNN	0.8722	2.6134	1.8397	0.9684	0.0607
CNN-SVR	0.9045	2.2587	1.4833	0.9764	0.0533
CNN-CBAM	0.9176	2.0979	1.3425	0.9793	0.0486
CNN-CBAM-SVR	0.9609	1.4448	1.0143	0.9901	0.0339
IAOA-CNN- CBAM -SVR	0.9919	0.6575	0.5253	0.9979	0.0153

**Fig 9:**
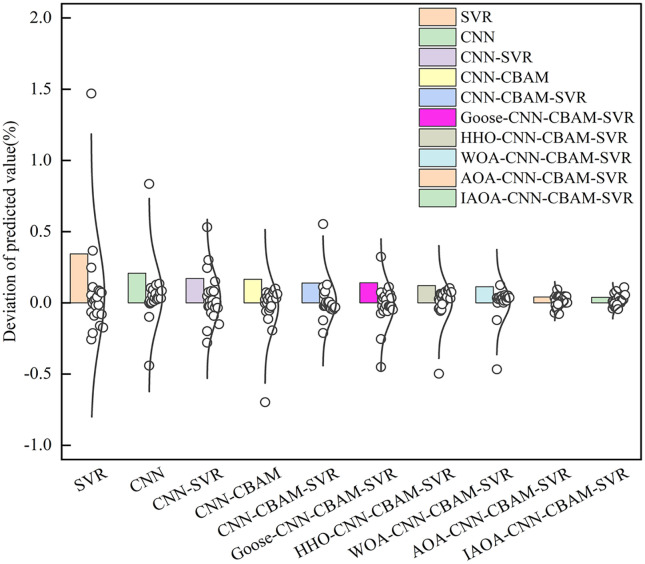
Normal distribution graph of model standard deviation.

Fig 9 shows the normal distribution graphs of the deviations for various models. As shown in Fig 9, the rectangular bars represent the standard deviation (SD) of each model, while the scattered points represent the differences between the actual values and the predicted values in the test set. The scattered points of each model follow a normal distribution. The SVR model exhibits a maximum divergence of 1.47, whereas the CNN model demonstrates a maximum deviation of 0.84. The standard deviations of the CNN-SVR, CNN-CBAM, and CNN-CBAM-SVR models are 0.1677, 0.1618, and 0.1384, respectively. Additionally, the standard deviations of the CNN-CBAM-SVR models optimized by Goose, HHO, WOA, AOA, and IAOA are 0.1367, 0.1188, 0.1107, 0.0411, and 0.0381, respectively. Especially, the scattered points are predominantly clustered around the center of the normal distribution for the AOA and IAOA optimized CNN-CBAM-SVR model. The IAOA-CNN-CBAM-SVR model exhibits the narrowest error band. This indicates that the deviation between the prediction and actual value was obviously reduced by IAOA. This is due to the IAOA employed good point set initialization instead of random initialization, thereby mitigating the uncertainty associated with random initialization and unequal spatial distribution of the population. Moreover, the IAOA implemented the Lévy rotation transformation approach to prevent duplicate individuals, broaden the search space, and effectively avoid local optima. The optimal hyperparameters of the CNN-CBAM-SVR model were obtained by IAOA, markedly improving the prediction accuracy.

**Fig 10:**
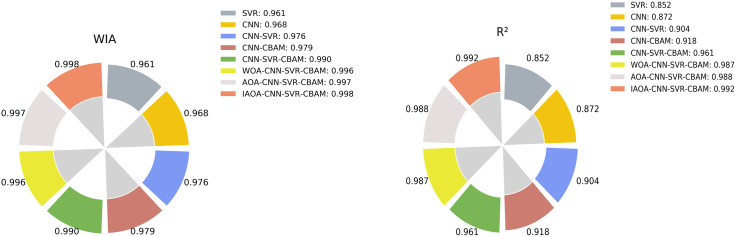
The circular rose diagram for R^2^ and WIA evaluation indicators.

Fig 10 shows the circular rose diagram of two statistical indicators of the coefficient of determination (R^2^) and the Wilmott Consistency Index (WIA) for various regression models. Both indicators show that the larger the value, the more accurate the model’s prediction will be. In Fig 10, as the rose diagram rotates clockwise, the indicator R² gradually increases. By comprehensively analyzing the WIA and R² indicators in the figure, the trajectory of the gradual optimization of the model performance can be clearly depicted: From WIA = 0.961 and R² = 0.852 of the basic SVR model to WIA = 0.998 and R² = 0.992 of the final IAOA-CNN-CBAM-SVR optimization model, an overall continuous upward development trend has been presented. Among them, the introduction of model fuses such as CNN-SVR has brought about the most significant initial performance improvement, with WIA increasing by 1.5% and R² increasing by 6.1%. The CBAM mechanism has achieved further fine enhancement, with WIA increasing by 0.3% and R² increasing by 1.4%. And the application of various intelligent optimization algorithms, especially IAOA, then a breakthrough leap in model accuracy was achieved, with a cumulative increase of 0.8% in WIA and a cumulative increase of 7.4% in R². The IAOA- CNN-CBAM-SVR model approaches the optimal values in both WIA and R² indicators, demonstrating a high degree of fitting ability and prediction consistency. The circular rose diagram reveals the marginal contribution characteristics of several models: the combination of CNN, CBAM, and SVR is the key driver for improvement prediction accuracy in small size samples. The proposed IAOA plays a crucial role in the process of approaching accuracy. This result not only reflects CNN-CBAM-SVR performance enhancement brought by the collaboration of DL and ML models but also highlights the core value of IAOA optimization in prediction air consumption of auxiliary nozzle with limited data, providing a practical and feasible path for high-precision and strongly robust regression model with small-scale samples.

Based on the previous comparative analysis, the following conclusion can be drawn. The superior performance of the proposed IAOA-CNN-CBAM-SVR model can be attributed to the complementary strengths of its components. The CNN-CBAM module acts as an effective feature extractor that captures complex nonlinear relationships among auxiliary nozzle structural parameters. The CBAM attention mechanism further enhances this process by emphasizing informative features and suppressing irrelevant information. Meanwhile, SVR provides strong generalization capability, particularly under small-sample conditions, due to its structural risk minimization principle. By combining deep feature extraction with a robust regression framework, the hybrid architecture mitigates the limitations of standalone machine learning or deep learning models. In addition, the Improved Archimedes Optimization Algorithm enables efficient hyperparameter tuning, helping the model avoid suboptimal parameter settings and improving prediction stability when training data are limited. Despite the promising results, several limitations should be acknowledged. The prediction accuracy may decrease when the auxiliary nozzle structural parameters fall outside the training data range, as the model primarily learns patterns within the sampled parameter space. In addition, although the dataset was designed using Latin Hypercube Sampling to ensure uniform coverage of the parameter space, the total number of samples remains relatively limited. This may lead to higher prediction uncertainty in regions where nonlinear interactions between parameters become more complex. Furthermore, the optimization process based on the IAOA algorithm increases the computational cost during the training stage, although this does not significantly affect the efficiency of the prediction stage.

### 5.5. Structure feature analysis

Understanding structure-air consumption relationships is crucial for extracting knowledge from the hybrid CNN-CBAM-SVR model. As an effective interpretability tool for “black box” models, the SHAP method can quantify the marginal contribution of input features to the output results and enhance the transparency and credibility of the model. Accordingly, an “importance” value is generated using the SHAP interpreter for visualizing analysis the contribution of each input feature to the output target of air consumption.

**Fig 11:**
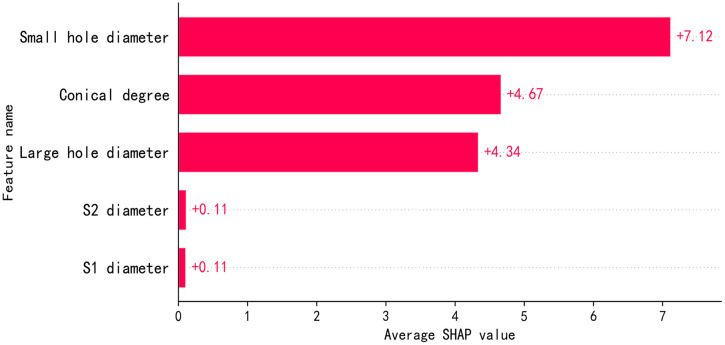
Summary diagram and importance diagram of auxiliary spray structure characteristics.

Fig 11 presents a summary diagram of the structural features of the auxiliary nozzle by the SHAP interpreter. The degree of influence of five structural features on air consumption was visualized in the summary diagram. The SHAP analysis results in the figure quantitatively reveal the influence intensity of each structural parameter on the output of the auxiliary nozzle air consumption prediction model. As seen in Fig 11, the SHAP value of small hole diameter(d1) is the highest at +7.12, indicating that its positive influence on output target is the most significant and it is the dominant feature. The SHAP values of conical degree(α) and large hole diameter(d2) are +4.67 and +4.34, respectively. Both the conical degree(α) and large hole diameter(d2) have a strong positive contribution, but their effect is less than the small hole diameter(d1). The SHAP values of inlet diameter (S1) and inlet diameter (S2) are +0.11, indicating that their influence on the output target is negligible.

**Fig 12:**
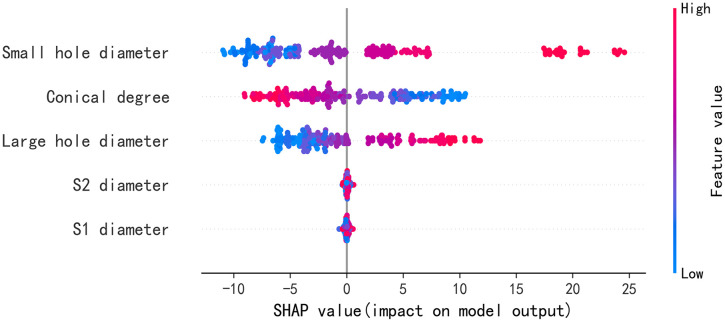
Summary diagram and importance diagram of auxiliary spray structure characteristics.

Fig 12 shows the bee swarm diagram for a theoretical framework of the SHAP values for the hybrid CNN-CBAM-SVR model. The color gradient represents the magnitude of the feature values according to the color bar. The point position along the horizontal *x* axis is determined by SHAP value and the vertical axis represents the feature. The color bar is standardized in accordance with the characteristic scope such that high characteristic value appears in red and low values appear in blue. Each scatter point in the graph represents the SHAP value of an instance. The darker the color, the larger the corresponding feature SHAP value. The further to the right the data point is, the greater the positive impact of the input variable on the prediction result of air consumption. The further to the left the data point is, the greater the negative impact of the input variable on the prediction result of air consumption. It can be seen from Fig 12 that the small hole diameter (d1) is the most significant contributing feature. The variation range of the SHAP value is from -10 to +25. When the SHAP value is between 15 and 25, this characteristic value is very high, indicating that the larger the value of this parameter, the more significant the influence on the prediction result. The large hole diameter(d2) maintains a stable positive correlation, and an increase in size will increase the air flow rate. Therefore, the influence of this feature on the prediction result is the same as that of the small hole diameter. However, the conical degree parameter(α) has a negative influence on air consumption. The SHAP value ranges from -10 to +10. In contrast, the SHAP values of the inlet diameters of S1 and S2 are both within ±1, having a minimal impact on the predicted output.

**Fig 13:**
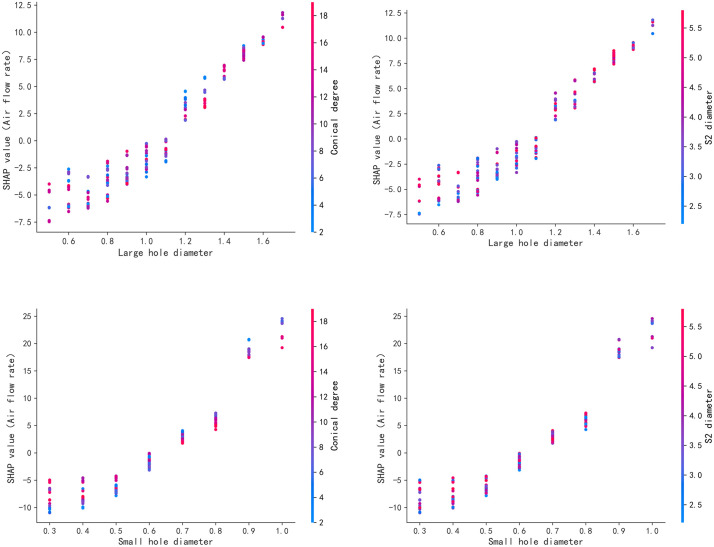
Auxiliary spray structure feature dependency diagram.

To further analyze the mutual influence relationship between two structural features, the feature dependency graph is shown in Fig 13. The vertical spread of SHAP values at a fixed characteristic values is a sign of interaction impacts with other characteristics in the model. From blue to red, it indicates that the characteristic value increases from small to large. A positive value indicates the output direction of increasing air consumption, and a negative value indicates the output direction of decreasing air consumption. It can be seen from the two figures above Fig 13 that there is a complex relationship among the diameter of the large hole, the taper, the diameter S2 of the air inlet and the air consumption. Both the left and right figures show an increasing form. As the horizontal coordinate moves to the right, the taper values are mostly smaller, while the S2 values of the inlet diameter are mostly larger. If only the diameter of the large hole is considered, within the range of d1 being 0.6 to 1 millimeter, the SHAP value of the output quantity is almost always less than 0, indicating that within this range, the diameter of the large hole has a relatively small impact on the output. Similarly, if we only look at the taper, when the range of α is between 10 and 20 degrees, the SHAP value of the output quantity is almost always less than 0, which indicates that within this range, the influence of the taper on the output is relatively small. It can be clearly seen from the two figures below Fig 13 that there is also a complex influence relationship among the diameter of the small hole, the taper, the inlet diameter S2 and the air consumption. The two pictures on the left and right also show an increasing form. If we only look at the diameter of the small hole, when the d2 range is between 0.3 and 0.6 mm, the SHAP value of the output quantity is almost always below 0, indicating that the diameter of the small hole has a relatively small impact on the output within this range.

The SHAP-based interpretability analysis also provides valuable insights for engineering design. By quantifying the contribution of each structural parameter to air consumption, the model helps identify the most influential design variables affecting energy usage in air-jet looms. For example, parameters with higher SHAP importance values indicate a stronger impact on air consumption and therefore should be prioritized during nozzle design optimization. These findings enable engineers to focus on adjusting critical structural features to achieve lower air consumption while maintaining stable weft insertion performance. Consequently, the proposed framework not only improves prediction accuracy but also supports practical decision-making in the design and optimization of auxiliary nozzles for enhanced energy efficiency.

## 6. Conclusion

To sum up, this study proposed and validated an explainable hybrid model, IAOA-CNN-CBAM-SVR, for quantitatively predicting the air consumption of auxiliary nozzle under small-sample conditions. The hybrid architecture integrated a CNN-CBAM feature extraction module with an SVR regression layer to enhance nonlinear representation and predictive accuracy. An Improved Archimedes Optimization Algorithm (IAOA) was developed to automatically optimize the model hyperparameters and mitigate premature convergence. Furthermore, SHAP analysis was employed to interpret the model outputs and quantitatively reveal the influence of each structural parameter on air consumption. The proposed framework not only achieved accurate prediction of nozzle air consumption but also provided valuable insights into the relationship between nozzle structure and energy efficiency in air-jet weaving systems.

The main contributions of this study are as follows:

1. Evaluation results using R², RMSE, MAE, TIC, and IA metrics demonstrated that the proposed model outperformed single-model approaches. Specifically, R² increased by 14% and 12% compared to SVR and CNN, respectively, while WIA increased by 4% and 3%. RMSE and MAE also decreased significantly, confirming the model’s predictive accuracy and reliability.
2. SHAP analysis revealed that the small hole diameter (d2) was the most sensitive feature influencing air consumption, followed by the large hole diameter (d1). The inlet diameters S1 and S2 contributed minimally, indicating they could be considered non-sensitive features. These findings provided mechanistic insights into how structural parameters affect air consumption and guided potential design improvements.
3. Compared with previous studies relying solely on SVR, RF, or CNN models, the proposed hybrid approach demonstrated better generalization under limited data conditions. The combination of deep learning, attention mechanisms, and metaheuristic optimization enabled both high predictive accuracy and interpretability, which is critical for practical applications in textile engineering.

Although the proposed IAOA-CNN-CBAM-SVR framework demonstrates strong predictive accuracy and interpretability, several limitations warrant acknowledgment. First, the proposed IAOA and feature extraction modules were validated on a limited set of experimental datasets involving auxiliary nozzles with multi-hole array configuration. As a result, the generalizability of the framework to other nozzle geometries or structural designs remains unverified. Second, the current model focuses on mapping multiple structural input features to a single output target- air consumption, leaving its capability for multiple input and multiple output (MIMO) nonlinear regression unexplored.

Building on the findings of this study, further investigation is warranted to assess the applicability of the proposed model to more complex auxiliary nozzle designs with high-dimensional structural input features. Moreover, reducing air consumption while maintaining high airflow velocity represents a critical trade-off in auxiliary nozzles. To address this challenge, the integration of multi-objective optimization strategies into the framework is essential, thereby enhancing its ability to simultaneously improve weft insertion efficiency and reduce energy consumption of auxiliary nozzles.
